# Padmashri Noshir Antia: Lotus of Indian plastic surgery

**DOI:** 10.4103/0970-0358.70714

**Published:** 2010-09

**Authors:** Shirish S. Daddi

**Affiliations:** Professor of Plastic Surgery and Burns. B.J.Medical College, and Sassoon General Hospitals, Pune. Sr. Plastic Surgeon, Jehangir Plastic Surgery Hospital, (Apollo Group), Pune. Aditya Birla Memorial Hospital, Pune. E-mail: daddi@vsnl.com

## EARLY DAYS

Born on February 8, 1922, Dr. Noshir Antia spent his childhood in Hubli and Belgaum Karnataka, and later his parents, father Hormasji and mother Soonamai, shifted to Mumbai. Dr. Antia completed his college education at Mumbai and Fergusson College, Pune. In 1945, he completed his medical graduation from Mumbai and served as Captain in British Army for two years.

## THE TURNING POINT

In 1947, he went to UK for higher studies and worked under the guidance of Sir Harold Gillies, the father of modern plastic surgery, from whom he imbibed his interest in plastic surgery. He obtained his Fellowship of the Royal College of Surgeons and stayed in UK for nine years. His close association with eminent plastic surgeons in UK and exposure to this new, challenging branch of plastic surgery inspired him to take up plastic surgery as his career.

## THE ICON

On his return from UK in 1958, he joined Jehangir Hospital in Pune, as a surgeon. I joined this hospital as a plastic surgeon in 1967 and I feel very fortunate to work in a place where this great person once lived and performed many operations and I feel thrilled in handling the same instruments that he touched. Senior theater staffs who worked with him used to narrate to me about the memories of his skill and dexterity. During his stay in Pune, he took keen interest in leprosy reconstructive surgery at Bandorwalla Leprosy Hospital in Kondwa. His involvement, in this special branch of plastic surgery, was soon recognized by the Government and he was invited to establish a Plastic Surgery Department in J. J. Hospital, Mumbai, which was inaugurated by Sir Harold Gilllies. Soon, this Department became famous all over the world and many renowned plastic surgeons started visiting Tata Dept. of Plastic Surgery and other plastic surgery centers in India for teaching and exchange of knowledge. Soon, burns, hand surgery and, leprosy surgery sections were added. This became an impetus for other institutes to upgrade their specialty sections. Prof. Antia’s first micro-vascular free flap in India is well documented.

Prof. Antia was associated as a founder member of numerous national and international associations and organizations and was honored with many prestigious awards. Dr. Noshir Antia was also instrumental in establishing the Association of Plastic Surgeons of India in 1957, and a few years later, the National Societies for Burns and Hand Surgery. He was a member of the Advisory Committee, Govt. of India, ICSSR and ICMR, national Rural Health Mission and many of his suggestions were accepted. After retirement, he dedicated his services towards community health and rural development. His association with community project at Parinche, Anna Hajare’s revolution, Equal Opportunity for the Handicapped, and Foundation for Medical Research in Community Health were simply inspirational. He was a founder member of the Association of Rural Surgeons of India, which has received global recognition and has today joined hands with the International Association of Rural Surgeons. In recognition of his many contributions to Health care and Plastic Surgery, he was awarded the Padma Shri in 1990 by the Government of India and the G. D. Birla International Award for Humanism in 1994. Both the APSI and its Maharashtra Chapter conferred on him the Life Time Achievement Award. He was awarded the Hunterian Professorship of the Royal College of Surgeons in 1962 and and the Clayton Memorial Lecturer of the Royal College of Surgeons of England, Maliniac Lecturer of the American Society of Plastic and Reconstructive Surgeons and the Pandalai and Sushrutha Oration of Association of Surgeons of India.

Very often, we hear that Indian contribution to scientific paper publication is lamentably low as compared to what it could be. Dr Antia is a stunning exception. A search of PubMed would reveal that he has 182 publications in peer reviewed journals - the first one appearing in 1955 and the last one in 2006! Many of his articles stand relevant to our practice even today. His sequence of publications also reveals the evolution of Dr. Antia, the man. The publications range from simple treatment vignettes (Use of Cephalic Vein in intravenous therapy in BMJ in 1955 - his first article in a peer reviewed journal) to Microsurgery to Economic models of healthcare. Dr. Antia’s last contribution was his autobiography -“A LIFE OF CHANGE” (Penguin Publication) and it is an extrordinary masterpiece.

His dedication, simple approach, and a deep sense of belonging and humility toward poor and down-trodden patients, particularly leprosy stigma sufferers gave him an un-paralleled status and international recognition. His firm and outspoken nature, sound ideas and innovations, and their successful implementation made him a distinguished and outstanding personality in the field of plastic surgery.

## ARNIE-‘SHINING’ SHADOW

Madam Arnie is a great lady. She has stood all the years by Dr. Antia’s side and contributed to a great extent in his success and achievements. She has been looking after most of Dr Antia’s projects, plans, offices, friends, visitors, staff all these years. Her total involvement is apparent in all the fields mentioned above.

## EPILOGUE

I was sitting humbly in front of him for an interview in his office at Plastic Surgery Department, J. J. Hospital, Mumbai, some time in early seventys. I was sent for selection to the post of Plastic Surgeon, Jehangir Hospital (Apollo Group), Pune & Birla Memorial Hospital, Pune. He said, he knew me and was aware of my work. He did not ask any questions. He advised me to continue my work, without waiting for new and sophisticated equipments. One can achieve equally good functional results with simple techniques and using basic instruments; he said. We progress if there is less! He told me to go round the department and to spend some time in the library. The interview was over.

Later, I met this great man during conferences where he watched my presentations. He rarely participated in open discussions, but during breaks he used to approach personally and always appreciated, gave encouragement and useful advice. Once he came to my Department, went around the wards, and saw my microsurgery work, which I performed without using operating microscope or loupes (I did not have either). Before leaving the Department, he gave his personal set of Keeler loupes to me. He was a very broad minded, kind, encouraging and helping person. I was humbled when he made an appreciative reference to my work in one of his orations he delivered in London. I cherish my memories of this beautiful ‘lotus of Indian Plastic Surgery!’

**Figure 1:**
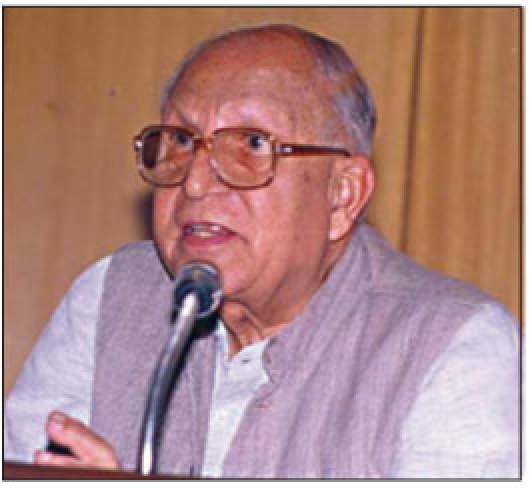
Padmashri Noshir Antia

